# Mineralogical Properties of a Refractory Tantalum-Niobium Slag and the Effect of Roasting on the Leaching of Uranium-Thorium

**DOI:** 10.3390/toxics10080469

**Published:** 2022-08-12

**Authors:** Min Huang, Ke Hu, Xiang Li, Yun Wang, Jinbo Ouyang, Limin Zhou, Zhirong Liu

**Affiliations:** 1 State Key Laboratory of Nuclear Resources and Environment, East China University of Technology, Nanchang 330013, China; 2 School of Chemistry, Biological and Materials Science, East China University of Technology, Nanchang 330013, China; 3 School of Nuclear Science and Engineering, East China University of Technology, Nanchang 330013, China

**Keywords:** tantalum-niobium slag, mineralogical properties, roasting, uranium, thorium, leach

## Abstract

In order to realize sustainable development, it is beneficial to explore an appropriate process to recover the radionuclides contained in tantalum-niobium slag. By micro-mineralogical analysis and roasting experiments, the effect of uranium-thorium leaching from a refractory tantalum-niobium slag is investigated. The uranium and thorium content in the slag is 2.26 × 10^3^ mg/kg and 7.84 × 10^3^ mg/kg, which have large recovery value. As the surface area and pore size of the slag are very small, the leaching agent cannot fully penetrate the particles. Various methods of characterization are used to analyze the mineralogical properties of roasted slag at different temperatures. The leaching ratio of U-Th is 90.84% and 96.62% at the optimum roasting temperature of 500 °C, which are about 39% and 27% higher than original samples. The oxidants Fe^3+^, O_2_ and Mn can also promote the conversion of insoluble U(IV) to soluble U(VI). Roasting reduces the content of organic C and S, thereby preventing reduction of U(VI), and increasing pore size as well as specific surface area also promote radionuclide leaching. Thus, the roasting method at 500 °C can destroy the surface wrapping structure of radionuclides, reduce the internal density of minerals, and improve uranium-thorium leaching ratio significantly. It is of great practical significance to reduce the radioactive hazard of waste tantalum-niobium slag and to strengthen the sustainable utilization of resources by suitable process improvement techniques.

## 1. Introduction

Both tantalum (Ta) and niobium (Nb) belong to group VB elements, which are transition metals with similar physicochemical properties [1,2,3]. They often exist as associated minerals in geological environment. Ta and Nb can form a thin oxide layer with high corrosion resistance on surface, so they are considered as excellent biologically inert materials [4]. Since the contraction of lanthanides, tantalum and niobium are related to each other, as well as occur in pairs. Furthermore, they form tantalates and niobates that contain large amounts of uranium (U) and thorium (Th) [5]. Tantalum-niobium minerals associated with uranium-thorium elements exist mainly in the form of multiple oxides. China is rich in reserves of uranium-thorium minerals, tantalum-niobium ores. Many minerals such as tantalum-niobium ore, biotite and monazite often contain certain amounts of radionuclides (especially U and Th) [6,7]. However, these rare heavy metal tailings have complex composition, low content of useful components, and are difficult to extract of heavy metals [8,9]. Additionally, many mines do not handle the waste properly after processing, leaving a great number of abandoned tailings [10]. The harmful substances rich in NH_4_^−^ and F^−^ in tailings cause secondary pollution [9]. This not only makes the utilization ratio of tailings low, but also endangers the ecological environment [11,12]. Thus, finding appropriate methods to solve the problem of comprehensively utilizing waste slag has become an urgent matter [13].

In recent years, the demand for Ta and Nb products have increased dramatically around the world, and the high-grade tantalum-niobium hard ores could no longer meet the market demand [14,15]. However, the decomposition of tantalum-niobium ore treated with H_2_SO_4_, HNO_3_ or HF is insufficient, resulting in a serious waste of resources. Moreover, the residual HF in the slag is volatile and easy to cause environmental problems [16]. Although tantalum-niobium resources in China are very rich, the ore grade is low, with many associated impurities, which leads to separate difficultly, low recovery ratio and poor occurrence state of useful components [17]. Tantalum-niobium ore undergoes a series of high-temperature as well as strong acid reactions in smelting process, during which the uranium-thorium remains in the slag and at a high content [9]. The risk of heavy metals contamination in mine waste cannot be ignored, especially when the tailings are affected by human activities [18]. After the smelting and processing of tantalum-niobium ore, the uranium-thorium elements in ore are largely retained in the tailings. These toxic elements are spread and remain by natural media, and eventually enter our bodies through the biological chain or biological cycle, posing a threat to human health [19,20]. Due to the radioactive nature of uranium and thorium, the extraction of radionuclides from mine solid waste is critical to environmental sustainability and human health [21,22].

With the development and utilization of nuclear energy, the market demand for nuclear fuel increased. If uranium and thorium in tantalum-niobium tailings can be recovered, the shortage of uranium resources in China can be alleviated. The first step in recovering uranium-thorium from tantalum-niobium slag is to extract the target elements from the hard-to-decompose slag, so that the radionuclides can be initially separated from the slag. The high intensity reaction occurring during smelting makes it difficult to leach uranium-thorium from tantalum-niobium tailings directly with acid. The original mineral structure is broken, resulting in a more complex composition [23]. Therefore, this work selected a refractory tantalum-niobium mine waste residue in south China as the research object, and analyzed the relevant mineralogical properties of tantalum-niobium slag by various mineralogical characterization methods. Additionally, the research elaborated the mineralogical microstructure characteristics of the slag, and explored the composition and distribution characteristics of uranium and thorium in the slag. It is one of the focuses of this work to explain the reason why uranium and thorium are difficult to leach from tantalum-niobium slag by micro-mineralogical perspective. Considering the presence of reductive organic C and S in the slag, and the possibility of some special inclusions to encapsulate radionuclides, so the slag was roasted. The conversion of insoluble U(IV) to U(VI) was promoted by roasting, thus improving the leaching ratio of radionuclides. The ultimate purpose of this research is to improve the recovery of uranium-thorium from tantalum-niobium slag and provide ideas for the treatment of waste slag in industrial applications.

## 2. Experiments and Methods

### 2.1. Instrumental Techniques

The characterization methods of tantalum-niobium slag are as follows. The microstructure of tantalum-niobium slag was observed by Scanning Electron Microscope (SEM) using a Nova-Nano SEM450 (FEI, Hillsboro, OR, USA). The types, content and distribution of the main elements in slag were detected by Energy Dispersive Spectroscopy (EDS) using a model x-max 50 spectrometer (Oxford Instruments, Abingdon, UK). Inductively Coupled Plasma-Optical Emission Spectrometer (ICP-OES) technique allows qualitative and quantitative analysis of the elements contained in slag samples. The content of various elements such as Ta, Nb, U, Th and Fe in slag, and the content of U as well as Th in the leaching solution during the detection experiments, were accurately detected by ICP-OES using an Avio200 type spectrometer (PerkinElmer, Waltham, MA, USA). In this work, the material structure of slag was analyzed by X-ray Diffractometer (XRD) of a D8-Advance device (Bruker, Karlsruhe, Germany). The types and content of elements in slag were detected by X-ray Fluorescence Spectrometry (XRF) using a model Axios-mAX spectrometer (PANalytical B.V., Almelo, The Netherlands). The elemental content of Fe and U in different valence states in slag was measured by X-ray Photoelectron Spectrometer (XPS) using a K-Alpha spectrometer (Thermo Fisher Scientific, Waltham, MA, USA). The microscale specific surface area and pore size of slag were characterized by specific surface area test (BET) using a BSD-PS1 type specific surface pore size analyzer (Beishide, Beijing, China). Also, the content of organic carbon and sulfur elements in slag was detected by organic carbon and sulfur analysis (TOC/TS) of a CS844 type analyzer (Laboratory Equipment Corporation, Livonia, MI, USA).

### 2.2. Roasting and Leaching Experiments

Considering the presence of reductive organic carbon and sulfur in tantalum-niobium slag, and the possibility of some special inclusions to encapsulate uranium and thorium, so the slag was roasted to improve the leaching ratio of radionuclides [24,25]. The changes of leaching ratio in slag before and after roasting was analyzed by ICP-OES technique to investigate the effect of roasting on leaching efficiency. In addition, the tantalum-niobium slag after roasting at 200–600 °C was analyzed to explore the optimal roasting temperature. In the roasting experiments, the insoluble U(IV) can be converted into soluble U(VI) by roasting, thus increasing the leaching ratio [26,27].

The tantalum-niobium slag should be pretreated before the experiments. The samples were dried in a blast drying oven at 50 °C for 24 h. The coarse samples were ground and sieved with a pulverizer and stored in a sealed bag. Samples with a particle size of −200 mesh (−75 μm) were selected for all experimental procedures. During roasting experiments, the pretreated slag was poured into the crucible, which was placed in muffle furnace at 200–600 °C (interval 100 °C) for 24 h. After cooling, the roasted slag was removed and stored in a sealed bag to keep dry and avoid contact between the sample and water vapor in the air, which may affect the experimental results.

In previous study, the effects of the type of acid used, the concentration of acid, leaching time, liquid-solid ratio and other conditions on the leaching ratio during the leaching process have been investigated [28]. In this work, we discussed the effect of roasting conditions on the leaching ratio of uranium-thorium from tantalum-niobium slag, using H_2_SO_4_ as leaching agent, and the original pH of the slag was 1.79.

A certain amount of −200 mesh raw slag and the samples roasted at 200–600 °C were respectively put into polytetrafluoroethylene beakers. The H_2_SO_4_ with the same concentration of 0.75 mol/L was added, the liquid-solid ratio was 10:1, the leaching temperature was 90 °C, and the magnetic stirrer was used for stirring and leaching for 4 h at the speed of 200 r/min. The leachate was obtained by filtration three times and analyzed by ICP-OES for U and Th content in the leachate. The leaching ratio was calculated by the equation (1), in this equation, *p_i_* (%) was the leaching ratio under condition i, *C_i_* (mg/L) was the element concentration in the leachate under condition i, *V* (L) was the volume of leaching agent, *W_i_* (mg/kg) was the content of uranium or thorium in the slag, *M* (kg) was the total mass of slag.

(1) $$p_{i}\;\left(\%\right)=\frac{C_{i}\times V}{W_{i}\times M}\times 100$$

## 3. Results and Discussion

### 3.1. Characterization Results of Mineralogical Properties of Slag

**Figure 1:**
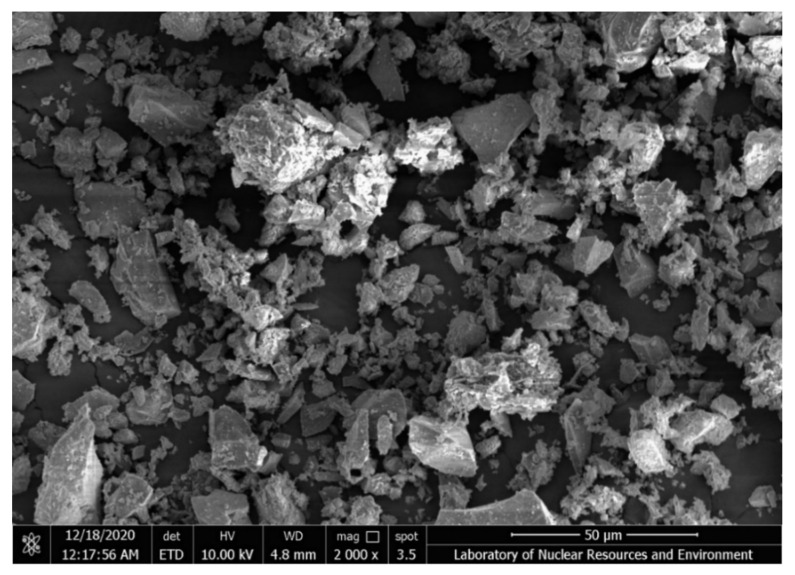
SEM image of tantalum-niobium slag.

**Figure 2:**
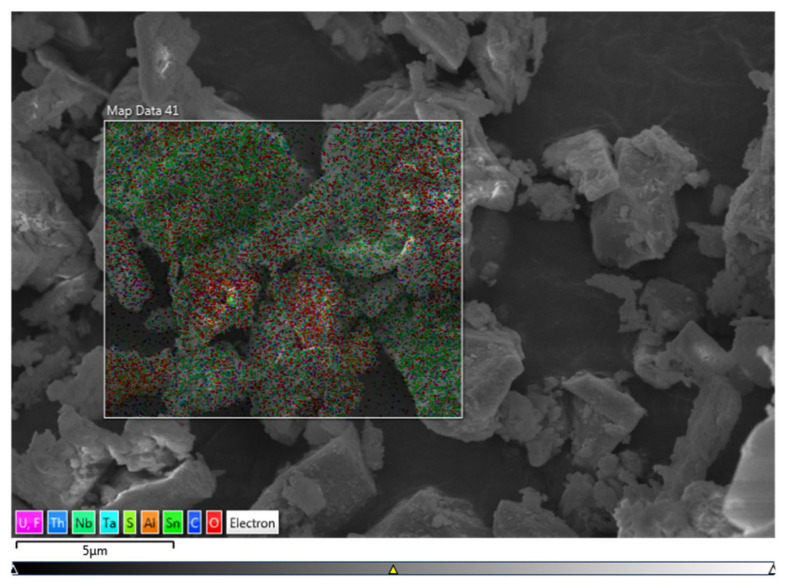
EDS image of tantalum-niobium slag.

The tantalum-niobium slag was analyzed by SEM and EDS, and the results were shown in Figure 1 and Figure 2.

Figure 1 was the SEM image of the tantalum-niobium slag at 2000×. It showed that many irregular particles in the slag, the surface between the particles was relatively dense, moreover, there were voids of different sizes between the particles.

The EDS results (Figure 2 and Table 1) of tantalum-niobium slag showed that the slag samples contain Al, F, Mn, Fe, Ta, Nb, Sn, U, Th, S and C. The content of F was 2.55% and S was 0.87%, which was due to the addition of large amounts of H_2_SO_4_ and HF during the smelting of tantalum-niobium ore, resulting in a small portion of F and S remaining in the slag. Besides uranium-thorium in the slag, there were also reducing substances such as metallic manganese, organic carbon, and sulfur, which could affect the redox atmosphere conditions in the leaching process. Furthermore, a certain amount of divalent iron was also present in the slag with a relatively uniform distribution, which can promote the leaching efficiency of U from slag. The addition of high concentrations of H_2_SO_4_ and HF destroyed the mineral structure, and made radionuclides enriched in slag.

### 3.2. Elemental Content in Tantalum-Niobium Slag

Table 2 showed that the tantalum-niobium slag was rich in Sn, additionally, the content of Ta, Nb and Fe was also relatively high, while the slag contained rare earth elements and radionuclides. These proved that the recovery of radioactive elements in tantalum-niobium slag was of significance.

In Table 3, the exact content of Ta, Nb, U, Th and Fe in the slag was obtained by ICP-OES analysis, with 2.26 × 10^3^ mg/kg of U and 7.84 × 10^3^ mg/kg of Th. The tantalum-niobium slag had a high content of U and Th, which was valuable for recycling. Additionally, the Fe element was rich to promote the oxidation of refractory U(IV) to soluble U(VI) and to improve the leaching efficiency. By recovering the uranium-thorium from tantalum-niobium slag, we can not only alleviate the shortage of nuclear fuel resources, but also reduce the pollution of slag on the surrounding environment.

### 3.3. Elemental Valence Analysis in Tantalum-Niobium Slag

The pretreated slag was taken and analyzed by XPS for the content of two different valence states of U and Fe in the slag, and the results were shown in Table 4.

The content of U and Fe in both valence states was in the form of divalent and trivalent compounds (Table 4). The content of ferrous compounds was 62.61%, and that of ferric compounds was 37.39%, which proved that most of Fe in slag existed in the form of ferric. Uranium in slag mainly existed in the form of tetravalent uranium dioxide, the content of U(IV) was 63.83%, and the content of soluble U(VI) was only 36.17%. In the original sample, the content of Fe^2+^ and U(IV) were higher than Fe^3+^ and U(VI). Although the existence of iron was beneficial to the subsequent leaching, the U(IV) was insoluble. Therefore, it was necessary to promote the conversion of U(IV) into soluble U(VI) by other means.

### 3.4. Specific Surface Area and Pore Size Analysis of Tantalum-Niobium Slag

**Figure 3:**
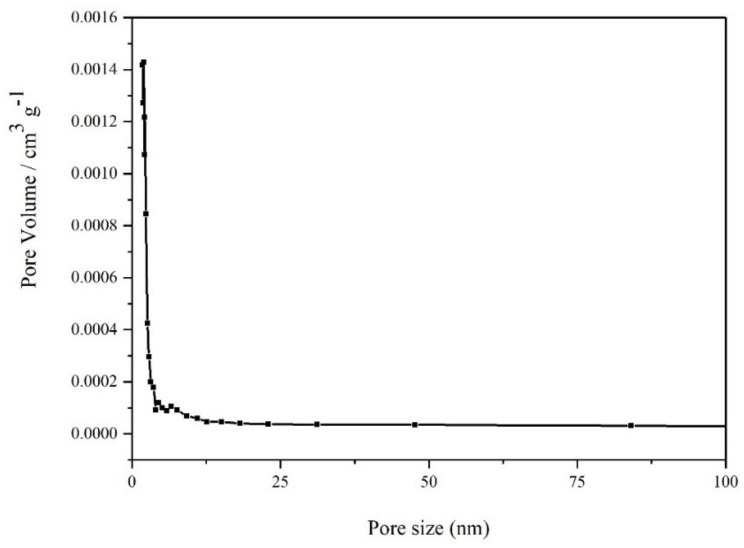
Pore size distribution of tantalum-niobium slag.

The Figure 3 showed that the pore size between the slag particles was small, and mainly distributed between 5 nm, thus the pore size in tantalum-niobium slag is mainly mesoporous (2–50 nm). This result was consistent with the data in the relevant literature.

There were many narrow pore sizes in the pore channels inside the slag, and the specific surface area, the average pore size as well as pore volume of the slag were relatively small (Table 5). The average pore size of the tantalum-niobium slag was 5.5453 nm, which verified that the pore size was mainly distributed around 5 nm, and the pore volume is 3.12 × 10^−3^ cm^3^/g. This was also consistent with the pore size distribution map of the slag. Nevertheless, the dense void distribution encased the radionuclides inside the slag, thus H_2_SO_4_ was not able to fully react with the slag particles. The following process was undertaken in order to investigate how roasting affects leaching, and to enhance the recovery effect by roasting experiments.

**Figure 4:**
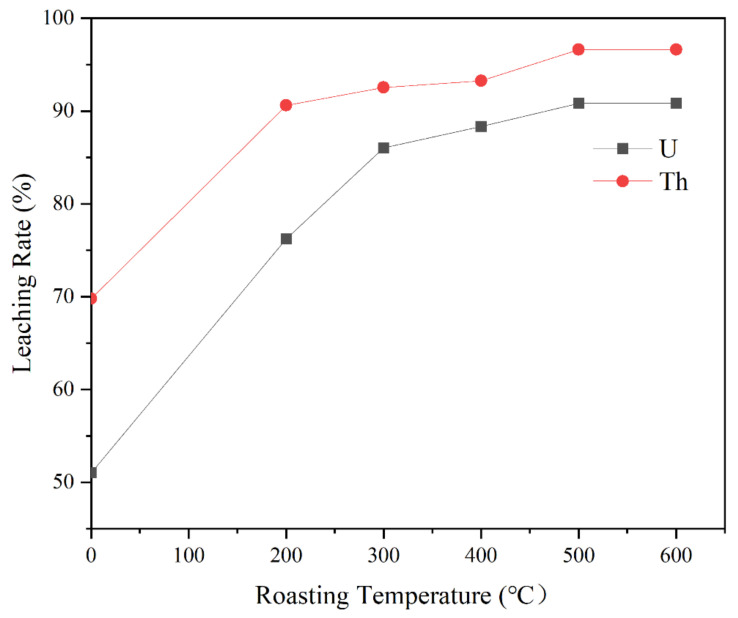
Effects of roasting temperature on uranium and thorium leaching.

The leaching ratio of U and Th of the raw slag was 51.86% and 69.64%. Similarly, the leaching ratio differed depending on the roasting temperatures (Figure 4). The leaching ratio of U and Th at 200 °C were 76.23% and 90.61%. The ratio increased with roasting temperatures from 200 to 500 °C. When the temperature was 500 °C, the leaching ratio reached 90.84% and 96.62%. When the temperature was from 500–600 °C, the ratio has no obvious change trend. Therefore, the roasting experiments could increase the leaching ratio of uranium-thorium. Moreover, 500 °C was chosen as the optimal roasting temperature to leach as much radionuclides as possible from tantalum-niobium samples.

### 3.5. Mechanism of the Effect of Roasting on the Leaching of Uranium-Thorium

Comparing the leaching results of raw slag and roasted samples, the leaching ratio of U increased about 39% as well as Th increased about 27% after roasting at 500 °C. This showed that roasting method had a facilitating effect on the extraction of radionuclides. Then, the slag roasted at different temperatures was analyzed by SEM, EDS, ICP-OES, TOC/TS, XRD, XPS and BET to explore the reasons for the increased leaching ratio of uranium-thorium from the slag.

The content of uranium-thorium in slag after roasting at different temperatures was measured by ICP-OES (Table 6).

The U-Th content in slag after roasting were increased. With the increase of roasting temperature, the uranium-thorium content in slag were gradually increased. Due to the decomposition of some components and water in slag during roasting, it reduced the overall quality of slag causing the raise of target elements content.

**Figure 5:**
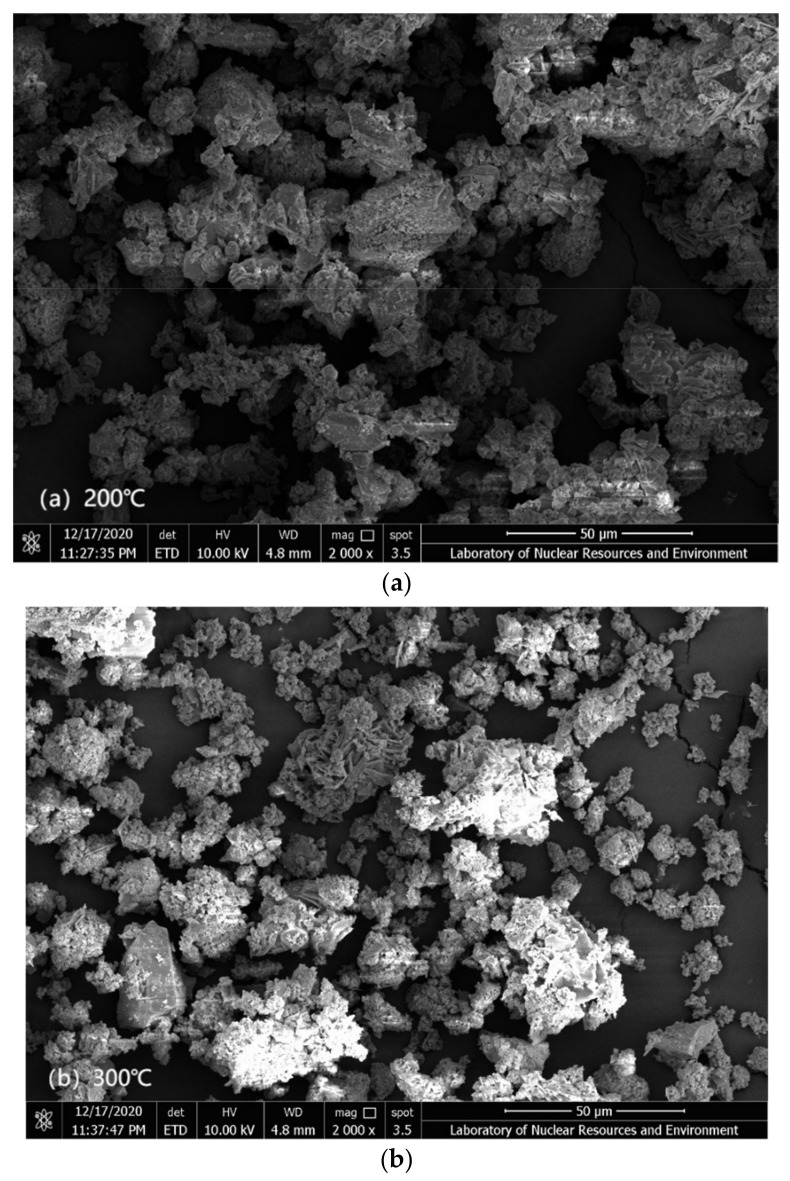

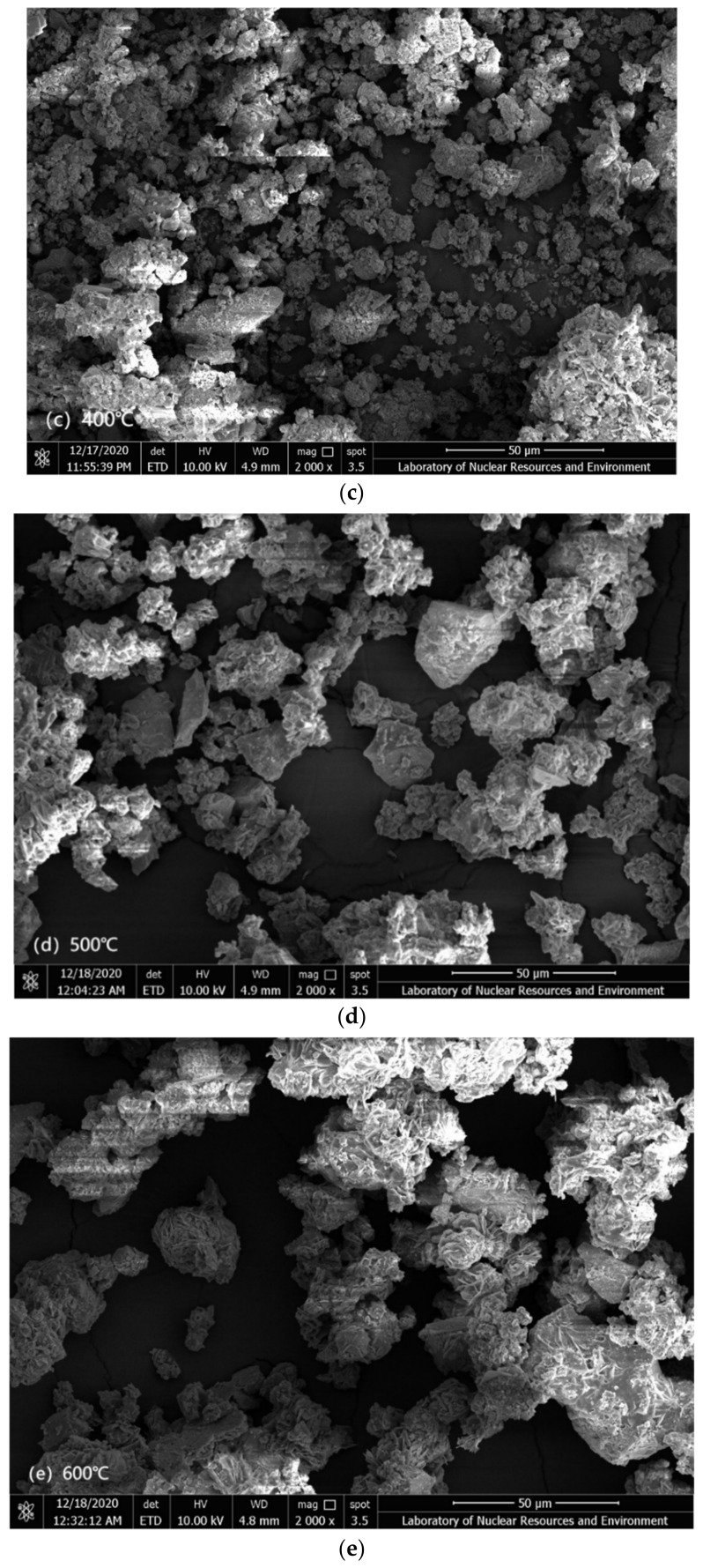
SEM images of tantalum-niobium slag roasted at (**a**) 200 °C; (**b**) 300 °C; (**c**) 400 °C; (**d**) 500 °C; (**e**) 600 °C.

Figure 5 showed the SEM images of the slag after roasting at 200–600 °C. As the roasting temperature increases, the slag presents irregular agglomeration and the voids between slag particles gradually increase, which was beneficial to the leaching of radionuclides. Under the condition of high temperature roasting, the surface impurities were dissolved and removed. The roasting method not only increased the particle void, but also dredged the pore size, thus promoting the elements leaching. When the temperature was 500 °C, the gap between slag particles was large enough and the dispersion was the best.

**Figure 6:**
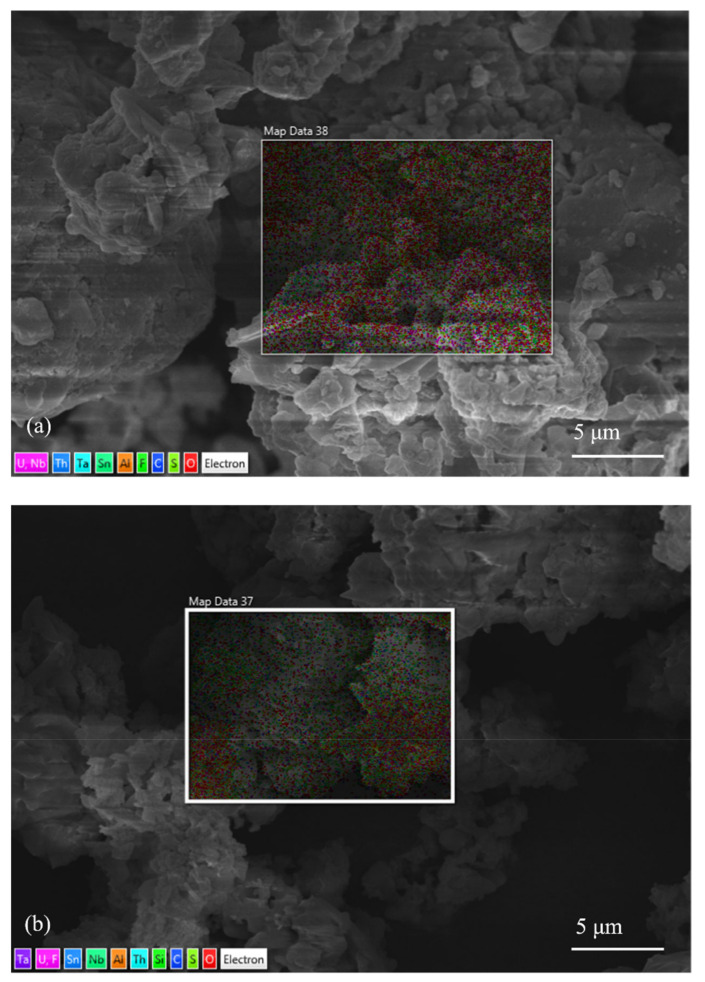

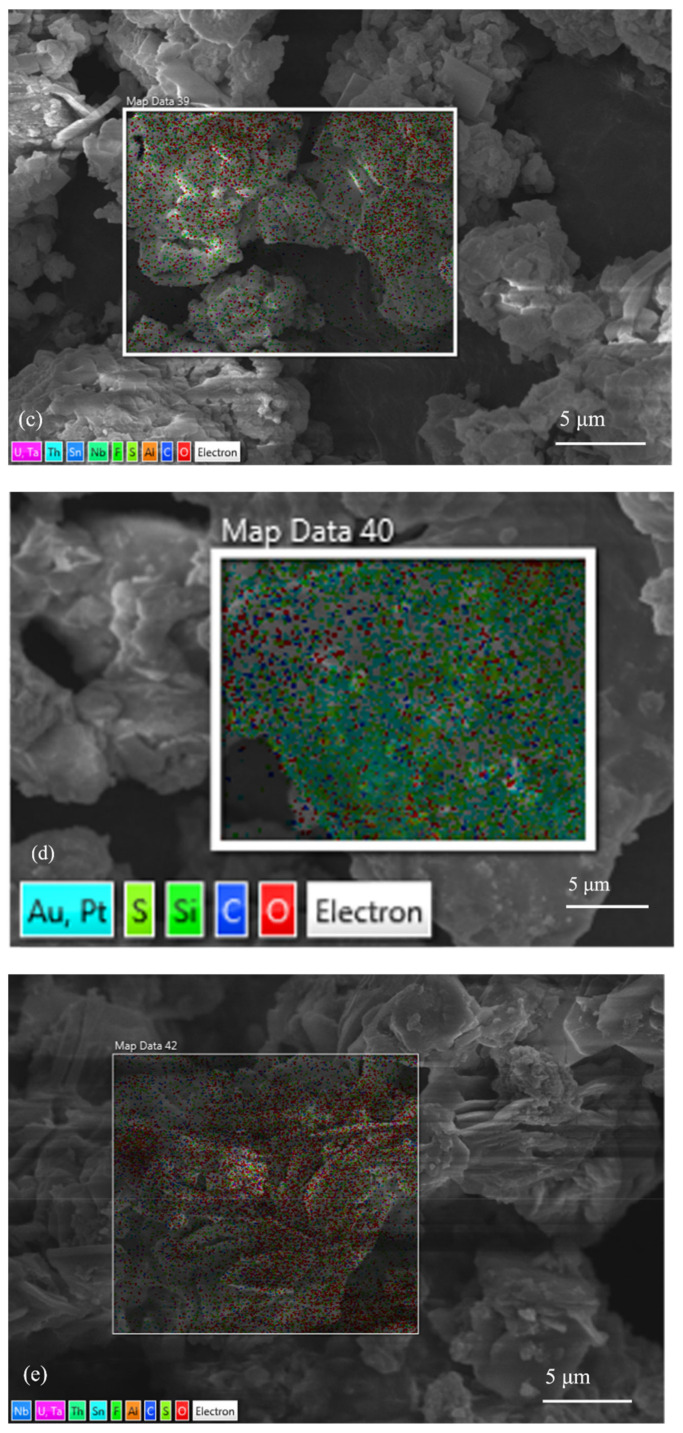
EDS results of tantalum-niobium slag roasted at (**a**) 200 °C; (**b**) 300 °C; (**c**) 400 °C; (**d**) 500 °C; (**e**) 600 °C.

Scanning results of slag roasted at 200–600 °C were shown in Figure 6.

After roasting at different temperatures, the content of C and S in slag changed, but the EDS technology did not measure the exact content of the elements. Thus, the organic carbon and sulfur analysis was used to obtain the exact content of organic carbon and sulfur elements.

Compared to the original samples, the organic C and S contents decreased at 200 °C. This indicated that the organic C and S within the slag reacted with oxygen in the air during the roasting process. Due to the reducibility of substances, the content of organic C-S reached the minimum at 500 °C and was conducive to leaching (Table 7). As reducing agents in the reaction, organic C and S restored the soluble U(VI) in the slag back to the insoluble U(IV). By roasting to raise the temperature, the reduction of U valence could be effectively controlled. Therefore, roasting could reduce the presence of organic C and S to improve the leaching ratio.

**Figure 7:**
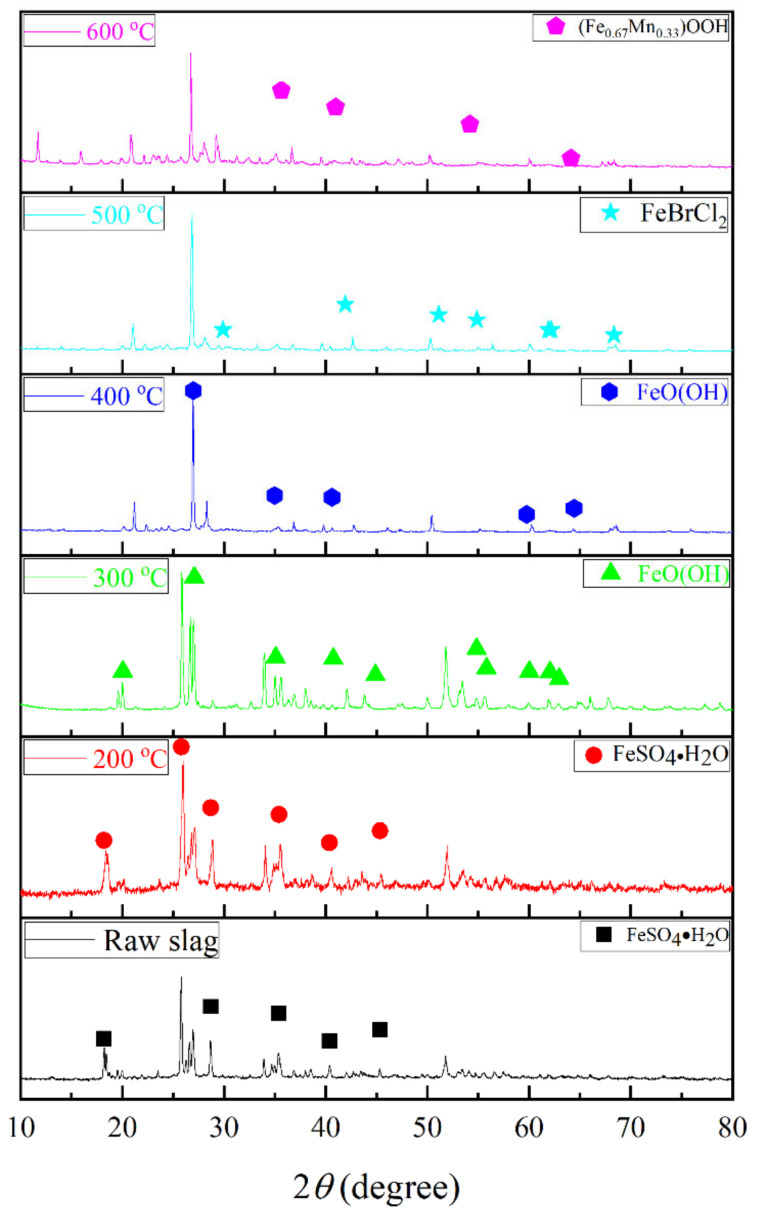
XRD of tantalum niobium slag.

The original slag and the 200–600 °C roasted slag were taken and analyzed by XRD for the composition of the physical phases, and the results were shown in Figure 7.

As shown in Figure 7, the XRD spectra of both the original slag and the 200 °C roasted slag matched with the standard PDF card for PDF #45-1364(FeSO_4_•H_2_O). The XRD spectra of the 300 °C and 400 °C roasted slag with high similarity to the standard PDF card for PDF #89-6096(FeO(OH)). The XRD spectra of the 500 °C and 600 °C roasted slag with high similarity to the standard PDF cards PDF #07-0284(FeBrCl_2_) and PDF #14-0557((Fe_0.67_Mn_0.33_)OOH). The comparison results confirmed that there were not only tantalum niobium oxide, but also bromine, iron, manganese and other compounds in the slag samples. Iron in the original slag mainly existed in the form of divalent salt compounds. Due to the complex composition of the tantalum-niobium slag and the variety of elements, the phase and the corresponding peaks of uranium-thorium were not detected from Figure 7. With the increase of roasting temperature, the divalent iron in slag gradually changed to higher valence. Therefore, the roasting method had oxidation effect on iron in tantalum-niobium slag.

The Fe^3+^ and U(VI) content in the tantalum-niobium slag reached 52.11% and 37.46% at 200 °C (Table 8). When the temperature was increased from 200 °C to 500 °C, the content of Fe^2+^ and U(IV) in the samples gradually decreased, and the Fe^3+^ and U(VI) reached a maximum of 89.43% and 66.79%. When the roasting temperature was 600 °C, the content of Fe^3+^ and U(VI) in the slag decreased compared with that at 500 °C. The above results illustrated that roasting could oxidize Fe^2+^ to Fe^3+^, and Fe^3+^ was used as an excellent oxidant in the reaction to facilitate the increase of the valence state of U(IV), this was conducive to leaching. 500 °C was the optimal temperature for roasting, which was consistent with the previous results.

**Figure 8:**
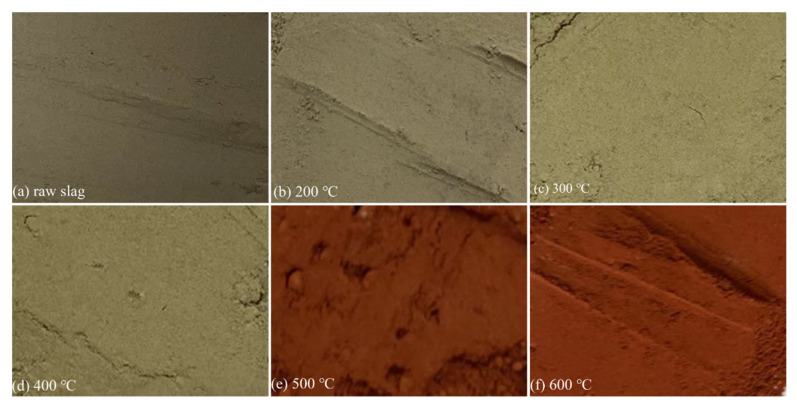
The images of (**a**) raw sample and roasted tantalum-niobium slag at (**b**) 200 °C; (**c**) 300 °C; (**d**) 400 °C; (**e**) 500 °C; (**f**) 600 °C.

Figure 8 showed that the raw slag was light gray and the color deepened after roasting with the temperature from 200 °C to 600 °C. As the roasting temperature reached 500 °C, the color of the slag changed to red. The results also indicated the conversion of divalent iron to trivalent iron in the slag, which corresponded to the results in the XPS. Thus, roasting had an oxidizing effect on the iron in the slag.

**Figure 9:**
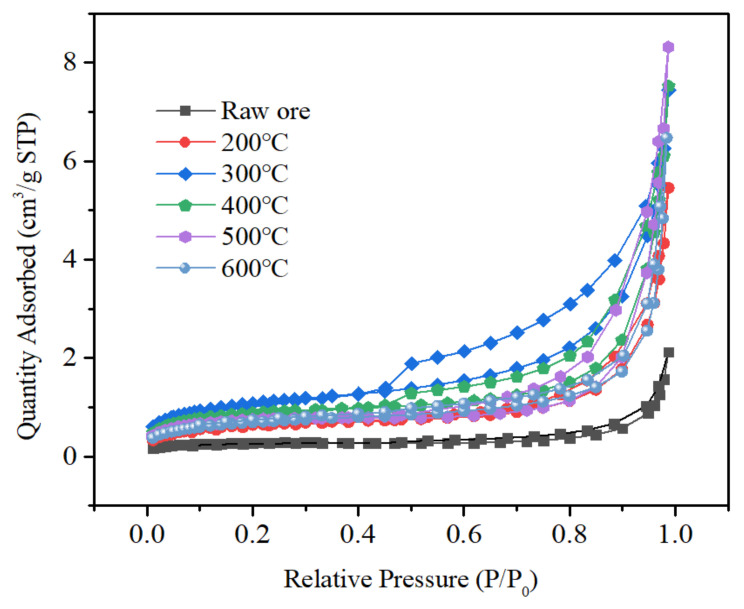
Adsorption and desorption isotherm of tantalum niobium slag to N_2_.

The curves of the raw and the roasted slag at five temperatures were type III adsorption isotherms (Figure 9). The N_2_ adsorption and desorption curves of slag after roasting at each temperature did not change much at relative pressures less than 0.8, and the adsorption of N_2_ by the slag increased significantly at relative pressures of 0.8–1.0. The maximum adsorption of slag was the highest at 500 °C roasting. Thus 500 °C can be chosen as the optimal temperature for roasting, corresponding to the previous results.

There are many narrow pore sizes in the pore channels inside the tantalum-niobium slag (Table 9). In addition, the specific surface area the pore size as well as volume of the slag are relatively small, which is in general agreement with the data in the relevant literature [29,30]. The pore size (9.2171 nm) and pore volume (12.720 × 10^−3^ cm^3^/g) of slag was maximum after roasting at 500 °C, which corresponded to the results of the maximum adsorption of the slag and verified the optimal roasting temperature. Roasting enlarged the pore size and gap of slag and increased the contact area between the leaching agent and the sample particles, allowing the leaching experiment to proceed more fully.

### 3.6. Morphological Mechanism Analysis of U

**Figure 10:**
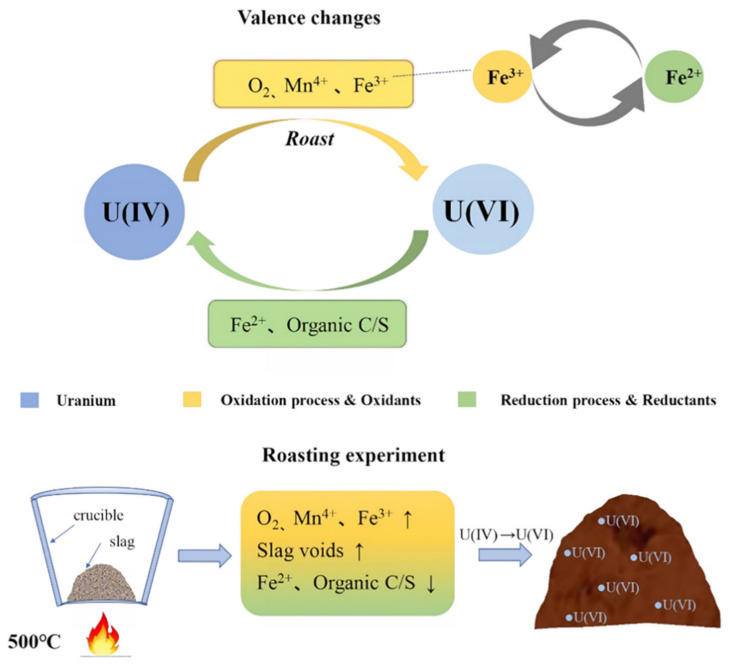
Morphological mechanism analysis of U.

Based on mineralogical characterization and roasting experiments, we can obtain the mechanism of morphological changes of uranium in the slag (Figure 10). In fact, the valence conversion of uranium involves the redox problem [31,32,33]. In the tantalum-niobium slag experiments, O_2_, Fe^3+^, and Mn^4+^ act as excellent oxidizing agents and influence the reaction atmosphere. These oxidants can promote the conversion of insoluble U(IV) to soluble U(VI), thus facilitating the U leaching in the subsequent stages [34]. However, due to the presence of organic carbon and sulfur in samples, the reduction of organic matter causes the conversion of U(IV) back to U(VI) again, limiting the extraction of U [35,36]. In the roasting experiments, by rising the temperature, the reaction intensity of oxidant is improved, while the content of organic carbon and sulfur is also reduced, reaching of a minimum at 500 °C, which improving the recovery effect. The treatment of samples by means of high-temperature roasting enables the removal of inclusions from the surface of the target elements and the unblocking of pore sizes. Therefore, roasting the slag at 500 °C can promote the leaching of radionuclides. The samples by means of high-temperature roasting removes impurities from the slag surface, strips the dense inclusions surrounding the target elements, and eliminates pore size obstruction. Therefore, calcination of slag at 500 °C can maximize the leaching of radionuclides.

## 4. Conclusions

The high concentration of H_2_SO_4_ and HF added during the smelting process destroys the mineral structure of tantalum-niobium ore, making U and Th in slag. The content of U and Th in the raw slag are 2.26 × 10^3^ mg/kg and 7.84 × 10^3^ mg/kg respectively, which have large recovery value. The initial leaching ratio of U and Th with H_2_SO_4_ as leaching agent is 51.86% and 69.64%. The average pore size of the slag particles is 5.5453 nm. There are many narrow pore channels between the particles, and the specific surface area is small, hindering the full contact between H_2_SO_4_ and slag. Besides U and Th in the slag, reducing substances such as Fe^2+^, organics and Mn can also affect the redox atmosphere conditions during leaching.

Considering the possibility of some special inclusions to encapsulate uranium and thorium, the slag is roasted to improve the leaching ratio. Various methods of characterization are used to analyze the mineralogical properties of roasted slag at different temperatures. The leaching ratio of U-Th is 90.84% and 96.62% at the optimum roasting temperature of 500 °C, which are about 39% and 27% higher than original samples. The valence of Fe changes from divalent to trivalent with increasing temperature (Fe^3+^ content increases by 52.04%), and the content of U(VI) reaches 66.79% (increases by 30.62%). The oxidants O_2_ and Mn can also promote the conversion of insoluble U(IV) to soluble U(VI). The organic C and S content is reduced after roasting, so this effectively prevented the conversion of U(VI) back to U(IV). The pore size of the slag increases from 5.5453 nm to 9.2171 nm, and the pore volume expands from 3.12 × 10^−3^ cm^3^/g to 12.72 × 10^−3^ cm^3^/g, which obviously facilitates the contact area between H_2_SO_4_ and samples. Consequently, the roasting method at 500 °C can destroy the surface wrapping structure of radionuclides, reduce the internal density of minerals, and improve uranium-thorium leaching ratio significantly. It is of great practical significance to reduce the radioactive hazard of waste tantalum-niobium slag and to strengthen the sustainable utilization of resources by suitable process improvement techniques.

## Figures and Tables

**Table 1:** Elemental percentage of tantalum-niobium slag by EDS.

Element	C	O	F	Al	Mn	Fe	Nb	Sn	Ta	Th	U	Total
Wt %	2.18	31.20	1.44	6.44	0.24	1.08	12.88	37.54	5.58	0.04	1.38	100
Atomic %	6.12	65.86	2.55	8.06	0.14	0.66	4.68	10.68	1.04	0.01	0.20	100

**Table 2:** Detection of composition content in tantalum-niobium slag by XRF.

Composition	Sn	Fe	U	Th	O	Al	S	Ta	Nb
Content (wt.%)	8.38	28.5	0.38	1.22	28.5	3.36	16.8	0.08	0.799
Composition	Nd	P	Sc	Si	Yb	Pb	Cr	Ca	Ti
Content (wt.%)	0.55	2.73	0.041	2.58	0.59	0.099	1.27	0.71	0.79

**Table 3:** ICP–OES element analysis diagram of tantalum niobium slag.

Ta-Nb Slag	U	Th	Ta	Nb	Fe
Content (mg/kg)	2.26 × 10^3^	7.84 × 10^3^	0.88 × 10^3^	2.01 × 10^3^	1.66 × 10^5^

**Table 4:** XPS element analysis diagram of tantalum-niobium slag.

Valence States	Fe^2+^ (%)	Fe^3+^ (%)	U(IV) (%)	U(VI) (%)
Original slag	62.61	37.39	63.83	36.17

**Table 5:** Tantalum-niobium slag performance parameters.

Materials	Pore Volume (cm^3^/g)	Average Pore Size (nm)	BET Surface Area (m^2^/g)
Ta-Nb slag	3.12 × 10^−3^	5.5453	99.9 × 10^−2^

**Table 6:** The content of U and Th in roasted slag.

Roasting Temperatures (°C)	U Content (mg/kg)	Th Content (mg/kg)
0	2.26 × 10^3^	7.84 × 10^3^
200	2.31 × 10^3^	7.88 × 10^3^
300	2.42 × 10^3^	7.96 × 10^3^
400	2.46 × 10^3^	8.04 × 10^3^
500	2.54 × 10^3^	8.10 × 10^3^
600	2.59 × 10^3^	8.12 × 10^3^

**Table 7:** TS/TOC element analysis diagram of tantalum niobium slag.

Elements (%)	Raw Slag	200 °C	300 °C	400 °C	500 °C	600 °C
C	0.1010	0.0924	0.0845	0.0698	0.0654	0.0762
S	0.0354	0.0321	0.0306	0.0241	0.0208	0.0256

**Table 8:** XPS element analysis diagram of tantalum-niobium slag after roasting.

Roasting Temperature (°C)	Fe^2+^ (%)	Fe^3+^ (%)	U(IV) (%)	U(VI) (%)
200 °C	47.89	52.11	62.54	37.46
300 °C	23.01	76.99	52.52	47.48
400 °C	13.13	86.87	38.96	61.04
500 °C	10.57	89.43	33.21	66.79
600 °C	19.11	80.89	45.55	54.45

**Table 9:** The tantalum-niobium slag after roasting performance parameters.

Roasting Temperature (°C)	Pore Volume (cm^3^/g)	Average Pore Size (nm)	BET Surface Area (m^2^/g)
200 °C	8.233 × 10^−3^	7.4207	2.2868
300 °C	11.619 × 10^−3^	7.3708	3.8298
400 °C	11.489 × 10^−3^	7.8396	3.1209
500 °C	12.720 × 10^−^^3^	9.2171	2.6696
600 °C	9.553 × 10^−3^	6.7793	2.4359

## Data Availability

Data is contained within the article. The data presented in this study are available in this published article.
